# Supplementary material for: Resolving the full spectrum of human genome variation using Linked-Reads
Source: Genome Res. 2019 Apr;29(4):635–45. doi: 10.1101/gr.234443.118 (PMC6442396; doi:10.1101/gr.234443.118)
Supplement: Supplemental Material [file supp_gr.234443.118_Supplemental_longranger-2.2.zip › longranger-2.2.tar.bz2!/lr2.2-public/lib/stan/cmdstan/stan/doc/api/html/windowed__adaptation_8hpp_source.html]

Stan: src/stan/mcmc/windowed\_adaptation.hpp Source File


|  |  |
| --- | --- |
|  | Stan  2.5.0  probability, sampling & optimization |


- Main Page
- Related Pages
- Namespaces
- Classes
- Files

- File List
- File Members

All Classes Namespaces Files Functions Variables Typedefs Enumerations Enumerator Friends Macros Pages

- src
- stan
- mcmc

windowed\_adaptation.hpp

Go to the documentation of this file.

1 #ifndef STAN\_\_MCMC\_\_WINDOWED\_\_ADAPTATION\_\_BETA

2 #define STAN\_\_MCMC\_\_WINDOWED\_\_ADAPTATION\_\_BETA

3

4 #include <ostream>

5 #include <string>

6

7 #include <stan/mcmc/base\_adaptation.hpp>

8

9 namespace stan {

10

11  namespace mcmc {

12

13  class windowed\_adaptation: public base\_adaptation {

14

15  public:

16

17  windowed\_adaptation(std::string name): estimator\_name\_(name) {

18  num\_warmup\_ = 0;

19  adapt\_init\_buffer\_ = 0;

20  adapt\_term\_buffer\_ = 0;

21  adapt\_base\_window\_ = 0;

22

23  restart();

24  }

25

26  void restart() {

27  adapt\_window\_counter\_ = 0;

28  adapt\_window\_size\_ = adapt\_base\_window\_;

29  adapt\_next\_window\_ = adapt\_init\_buffer\_ + adapt\_window\_size\_ - 1;

30  }

31

32  void set\_window\_params(unsigned int num\_warmup,

33  unsigned int init\_buffer,

34  unsigned int term\_buffer,

35  unsigned int base\_window,

36  std::ostream\* e = 0) {

37

38  if (num\_warmup < 20) {

39  if (e) {

40  \*e << "WARNING: No " << estimator\_name\_ << " estimation is" << std::endl;

41  \*e << " performed for num\_warmup < 20" << std::endl << std::endl;

42  }

43  return;

44  }

45

46  if (init\_buffer + base\_window + term\_buffer > num\_warmup) {

47

48  if (e) {

49  \*e << "WARNING: The initial buffer, adaptation window, and terminal buffer" << std::endl;

50  \*e << " overflow the total number of warmup iterations." << std::endl;

51  }

52

53  num\_warmup\_ = num\_warmup;

54  adapt\_init\_buffer\_ = 0.15 \* num\_warmup;

55  adapt\_term\_buffer\_ = 0.10 \* num\_warmup;

56  adapt\_base\_window\_ = num\_warmup - (adapt\_init\_buffer\_ + adapt\_term\_buffer\_);

57

58  if(e) {

59  \*e << " Defaulting to a 15%/75%/10% partition," << std::endl;

60  \*e << " init\_buffer = " << adapt\_init\_buffer\_ << std::endl;

61  \*e << " adapt\_window = " << adapt\_base\_window\_ << std::endl;

62  \*e << " term\_buffer = " << adapt\_term\_buffer\_ << std::endl << std::endl;

63  }

64

65  return;

66

67  }

68

69  num\_warmup\_ = num\_warmup;

70  adapt\_init\_buffer\_ = init\_buffer;

71  adapt\_term\_buffer\_ = term\_buffer;

72  adapt\_base\_window\_ = base\_window;

73  restart();

74

75  }

76

77  bool adaptation\_window() {

78  return (adapt\_window\_counter\_ >= adapt\_init\_buffer\_)

79  && (adapt\_window\_counter\_ < num\_warmup\_ - adapt\_term\_buffer\_)

80  && (adapt\_window\_counter\_ != num\_warmup\_);

81  }

82

83  bool end\_adaptation\_window() {

84  return (adapt\_window\_counter\_ == adapt\_next\_window\_)

85  && (adapt\_window\_counter\_ != num\_warmup\_);

86  }

87

88  void compute\_next\_window() {

89

90  if (adapt\_next\_window\_ == num\_warmup\_ - adapt\_term\_buffer\_ - 1) return;

91

92  adapt\_window\_size\_ \*= 2;

93  adapt\_next\_window\_ = adapt\_window\_counter\_ + adapt\_window\_size\_;

94

95  if (adapt\_next\_window\_ == num\_warmup\_ - adapt\_term\_buffer\_ - 1) return;

96

97  // Bounday of the following window, not the window just computed

98  unsigned int next\_window\_boundary = adapt\_next\_window\_ + 2 \* adapt\_window\_size\_;

99

100  // If the following window overtakes the full adaptation window,

101  // then stretch the current window to the end of the full window

102  if (next\_window\_boundary >= num\_warmup\_ - adapt\_term\_buffer\_) {

103  adapt\_next\_window\_ = num\_warmup\_ - adapt\_term\_buffer\_ - 1;

104  }

105

106  }

107

108  protected:

109

110  std::string estimator\_name\_;

111

112  unsigned int num\_warmup\_;

113  unsigned int adapt\_init\_buffer\_;

114  unsigned int adapt\_term\_buffer\_;

115  unsigned int adapt\_base\_window\_;

116

117  unsigned int adapt\_window\_counter\_;

118  unsigned int adapt\_next\_window\_;

119  unsigned int adapt\_window\_size\_;

120

121  };

122

123  } // mcmc

124

125 } // stan

126

127 #endif

base\_adaptation.hpp

stan::mcmc::windowed\_adaptation::num\_warmup\_

unsigned int num\_warmup\_

**Definition:** windowed\_adaptation.hpp:112

stan::mcmc::windowed\_adaptation::windowed\_adaptation

windowed\_adaptation(std::string name)

**Definition:** windowed\_adaptation.hpp:17

stan::mcmc::windowed\_adaptation::end\_adaptation\_window

bool end\_adaptation\_window()

**Definition:** windowed\_adaptation.hpp:83

stan::mcmc::windowed\_adaptation

**Definition:** windowed\_adaptation.hpp:13

stan::mcmc::windowed\_adaptation::adapt\_base\_window\_

unsigned int adapt\_base\_window\_

**Definition:** windowed\_adaptation.hpp:115

stan::mcmc::windowed\_adaptation::adapt\_window\_size\_

unsigned int adapt\_window\_size\_

**Definition:** windowed\_adaptation.hpp:119

stan::mcmc::windowed\_adaptation::adaptation\_window

bool adaptation\_window()

**Definition:** windowed\_adaptation.hpp:77

stan::mcmc::windowed\_adaptation::adapt\_init\_buffer\_

unsigned int adapt\_init\_buffer\_

**Definition:** windowed\_adaptation.hpp:113

stan::mcmc::windowed\_adaptation::set\_window\_params

void set\_window\_params(unsigned int num\_warmup, unsigned int init\_buffer, unsigned int term\_buffer, unsigned int base\_window, std::ostream \*e=0)

**Definition:** windowed\_adaptation.hpp:32

stan::mcmc::windowed\_adaptation::adapt\_term\_buffer\_

unsigned int adapt\_term\_buffer\_

**Definition:** windowed\_adaptation.hpp:114

stan::mcmc::windowed\_adaptation::adapt\_next\_window\_

unsigned int adapt\_next\_window\_

**Definition:** windowed\_adaptation.hpp:118

stan::mcmc::windowed\_adaptation::restart

void restart()

**Definition:** windowed\_adaptation.hpp:26

stan::mcmc::windowed\_adaptation::compute\_next\_window

void compute\_next\_window()

**Definition:** windowed\_adaptation.hpp:88

stan::mcmc::base\_adaptation

**Definition:** base\_adaptation.hpp:8

stan::math::e

double e()

Return the base of the natural logarithm.

**Definition:** constants.hpp:86

stan::mcmc::windowed\_adaptation::estimator\_name\_

std::string estimator\_name\_

**Definition:** windowed\_adaptation.hpp:110

stan::mcmc::windowed\_adaptation::adapt\_window\_counter\_

unsigned int adapt\_window\_counter\_

**Definition:** windowed\_adaptation.hpp:117


---

[ Stan Home Page ]

*© 2011–2014,
Stan Development Team.*
